# Case report: Open replacement of incomplete semi-circular traumatic ruptures of the ascending and descending aorta

**DOI:** 10.1186/s13019-016-0485-0

**Published:** 2016-07-16

**Authors:** Miroslawa Mytsyk, Martin T. R. Grapow, Jasmin Shahinian, Markus Maurer, Lorenz Gurke, Friedrich S. Eckstein

**Affiliations:** Division of Cardiac Surgerz, University Hospital of Basel, Basel, Switzerland; Department of Anesthesia, University Hospital of Basel, Basel, Switzerland; Division of Vascular Surgery, University Hospital of Basel, Basel, Switzerland

**Keywords:** Traumatic rupture of the thoracic aorta, CT-scan, TOE, Replacement of the ascending aorta

## Abstract

An incomplete traumatic rupture of the ascending aorta is a rare but life-threatening condition. Hence, the assessment of the extent of the injury prior to therapy is crucial. We report a case of a 50-year-old male with traumatic aortic rupture who underwent emergency surgery after the evaluation of computed tomography scan (CT-scan). The surgical treatment involved replacement of the ascending aorta and stent implantation in descending aorta due to its covered rupture.

## Background

Traumatic aortic rupture is a life-threatening condition associated with high mortality and morbidity requiring immediate surgical treatment. The involvement of the thoracic aorta displays a potential fatal injury causing death in 75-90 % of cases at the time of the injury [1, 2]. Bending stress of aorta occurs as it is flexed over the left pulmonary artery and left bronchus. A recent analysis of traumatic aortic injury elucidated that torsion is a possible mechanism in an ascending aortic injury, which occurs above the aortic valve through rotational displacement of the heart at the time of the impact [3, 4]. In order to improve the outcome of thoracic aortic injury and the degree of multi-organ damage, it is crucial to accurately evaluate the priority of treatment [5].

Thoracic aortic rupture is a devastating injury and it rarely occurs as a sole traumatic entity. The acknowledgment of concomitant thoracic, abdominal, head injuries, and fractures after thoracic aortic rupture is of paramount importance [6]. Algorithms for the diagnosis and treatment of traumatic thoracic aortic injury have undergone changes in recent years [7].

Endovascular surgery of aortic injury became the treatment of choice, especially for patients with associated severe injuries and risk of bleeding [8]. Conventional surgical treatment is always indicated for young patients with stable hemodynamic condition, low risk of bleeding and when surgery can be delayed for several hours [9].

## Case presentation

A 50-year-old patient was brought to the emergency room after a failed suicide attempt by jumping from a 12 m high Psychiatric Clinic building, where he received treatment for his depression.

On admission, the intubated patient had a GCS of 3 points and was in certain unstable hemodynamic state with low inotropic support. The clinical evaluation showed multiple rib fractures and suppressed breath sounds on the right side. The vital signs at this point showed blood pressure of 80/55 mmHg without vasopressors, pulse of 100 bpm, oxygen saturation of 100 % on ventilation and temperature of 33.7 °C. External bleeding was not observed. Central and peripheral pulses in all four extremities were palpable but weak. No abdominal pain or tenderness was observed. The blood analysis showed following results: hemoglobin 144.0 g/l, hematocrit 0.41/l, erythrocytes 4.24 10 s^9^/l.

**Fig. 1:**
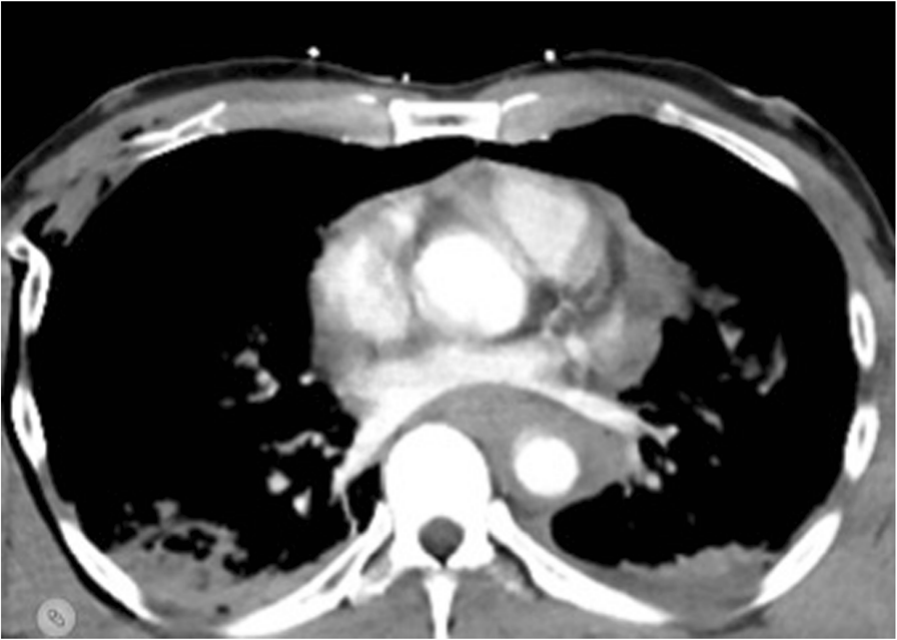
CT Thorax after contrast dye injection showing the rupture of ascending aorta. Arrow highlights aortic wall

A CT-scan according to polytrauma scheme (cranial-cervical-thoracic-abdomen-pelvis-leg) was immediately performed (Fig. 1), which showed disseminated cerebral bleedings near the cortex and interventricular hemorrhages associated with contusions, and small hemorrhage in the corpus callosum. Traumatic, semicircular and covered rupture of the middle of the ascending aorta appeared, the descending aorta presented a covered rupture, too. In addition, a moderate pericardial effusion of 7 mm was observed. No further dissection of the aortic root, ascending aorta or aortic arch was detected. Further, ventral pneumothorax on both sides with moderately displaced rib fractures and longitudinal fracture of the sacrum from the left side was detected.

### Treatment

**Fig. 2:**
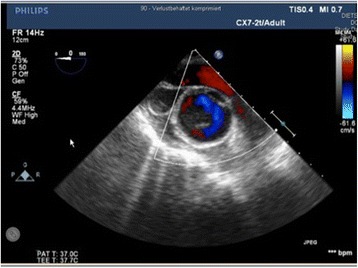
TOE. Ascending aorta. Arrow highlights aortic wall

**Fig. 3:**
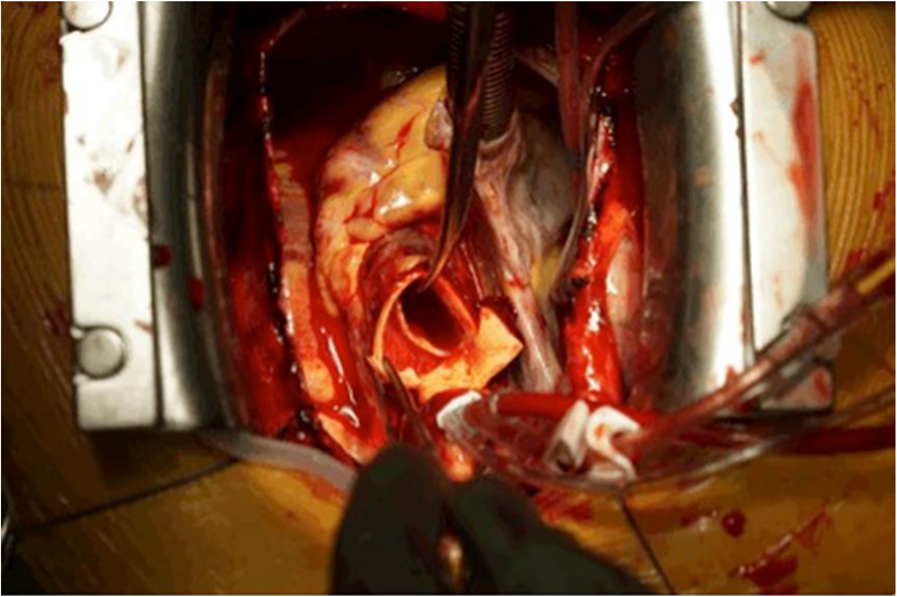
The arrow shoes the site of the rupture

The therapy involved interdisciplinary surgical treatment involving cardiac, vascular, and trauma surgical teams. At first, a Thoracic Stent Graft (W. L. Gore & Associates, USA, TAG 28/15) was introduced into the descending aorta via the femoral artery in Seldingers Technique to cover the rupture of the descending aorta performed by vascular surgeons. The intraoperatively performed transesophageal echocardiography (TOE) confirmed the incomplete rupture of the ascending aorta (Fig. 2). Thereafter a median sternotomy was performed and extracorporal circulation was installed after aortic cannulation of the proximal aortic arch and standard two-stage venous cannulation. After cross-clamping of the ascending aorta distally to the rupture site crystalloid cardioplegia was administered followed by blood cardioplegia. Intraoperative assessment of the aortic injury confirmed traumatic aortic rupture at the mid region of the ascending aorta. The aortic wall was incompletely ruptured with the rupture being covered by the pulmonary artery (Type II according to Goarin [10]). As a lucky consequence there was no blood effusion into the pericardium (Fig. 3). The ruptured aortic segment was replaced using a Gelweave 26 mm (Vascutek/Terumo, Great Britain) aortic straight graft. Thereafter the cardiovascular operation was finished in an uneventful routine way.

Further, an external fixation of symphysis with left sacrum and right sacroiliac joint (SIJ) was performed followed by chest ventrolateral wall stabilization from the 4 - 6th ribs. After 20 days of intensive care, the patient was transferred to the cardiac surgical ward. During the postoperative course the patient developed pneumonia, which was successfully treated with antibiotics. There were no surgery related complications. The postoperative follow-up CT control imaging showed a good result. The patient was discharged 21 days after the surgery to further rehabilitation under psychiatric control.

## Conclusion

This case report describes the management of an incomplete aortic semi-circular traumatic rupture caused by failed suicide attempt due jumping from 12 m height, by replacement of the ascending aorta and stent implantation in descending aorta. Traumatic aortic injury is a lesion extending from the intima to the adventitia. The region subjected to the greatest strain involves the area around the isthmus where the relatively mobile thoracic aorta joins the fixed aortic arch and the insertion of the arterial ligament. This region is the rupture site in 80 % according to pathological series and up to 95 % in clinical series. Lesions of the thoracic aortic wall occur in transverse manner, either segmental (55 %) or circular (45 %) like in our case. The CT-scan presents an excellent imaging tool to assess the extent of aortic trauma and is used as a standard diagnostic method in cases of multiple trauma and aortic dissection [11–13]. Other imaging tools used in the diagnosis of aortic rupture involve TOE and angiography [14, 15]. TOE has a high degree of sensitivity [~100 %] and 98 % specificity in the detection of aortic injury [16]. TOE guidance is considered appropriate in endovascular thoracic aortic procedures for monitoring, procedural guidance, and/or endovascular graft leak detection (Class IIa, Level of Evidence: B) [17]. CT-Scan is the standard approach in diagnosis of aortic rupture. However, if CT-scan is not conclusive like in our case additional imaging like TOE is useful to enhance detection of aortic injury.

## Abbreviations

CT-scan, computer tomography scan; GCS, the Glasgow coma scale; TOE, transesophageal echocardiography.
